# Destructive Upper Airway Disease from Eosinophilic Granulomatosis with Polyangiitis (EGPA): The Very First Case

**DOI:** 10.1155/2019/6173869

**Published:** 2019-05-23

**Authors:** Biplab Saha, Aditi Saha, Fernanda Cordeiro-Rudnisky, Boris Shkolnik, Scott Beegle

**Affiliations:** ^1^Division of Pulmonary and Critical Care Medicine, Albany Medical Center, Albany, NY, USA; ^2^Department of Medicine, Saint Barnabas Medical Center, Livingston, NJ, USA; ^3^Department of Pathology, Albany Medical Center, Albany, NY, USA

## Abstract

Eosinophilic granulomatosis with polyangiitis (EGPA) is a multisystem vasculitic disorder that predominantly affects medium- and small-sized blood vessels. EGPA belongs to a group of vasculitides known as anti-neutrophil cytoplasmic antibody- (ANCA-) associated vasculitis (AAV). Upper airway involvement is seen in all ANCA-associated vasculitides, but destructive upper airway disease has never been reported in patients with EGPA. We report the first case of erosive chondritis and saddle nose deformity in a 50-year-old patient suffering from EGPA.

## 1. Introduction

Eosinophilic granulomatosis with polyangiitis (EGPA), previously known as Churg-Strauss syndrome, is a multisystem vasculitic disorder that predominantly affects small-sized blood vessels. EGPA belongs to a group of vasculitides known as anti-neutrophil cytoplasmic antibody- (ANCA-) associated vasculitis (AAV). The other members of this category include granulomatosis with polyangiitis (GPA), formerly known as Wegener's granulomatosis and microscopic polyangiitis (MPA). Although these diseases share many common features, EGPA is unique in its presentation and usually follows a stepwise disease progression characterized by a prodromal period followed by an eosinophilic and vasculitic phases [1]. Upper airway involvement is seen in all ANCA-associated vasculitides, but destructive upper airway disease has never been reported in patients with EGPA. Here, we present the first case of saddle nose deformity due to erosive chondritis in a patient suffering from EGPA.

## 2. Case Presentation

A 50-year-old Caucasian female with a history of difficult-to-control asthma since 1994 and chronic rhinitis presented to the hospital with severe jaw and ear pain in late February of 2009. She had been suffering from intermittent pain for a few months and underwent bilateral myringotomy tube placement about a month prior for recurrent otitis media with some benefit. The pain was distributed over the rami of the mandible bilaterally with radiation to her ears. She denied any fever, night sweats, weight loss, purulent nasal discharge, odynophagia, dysphagia, or shortness of breath. No significant history of travel or sick contact including contact to TB patients. The patient was a past smoker with 15 pack year history of smoking.

Vital signs on admission showed a BP of 101/63 mm·Hg, pulse of 105 beats/minute, temperature of 97.9 F, respiratory rate of 18 breaths/min, and SPO_2_ of 96% on room air. Physical examination revealed a patient in moderate distress. Bilateral tenderness was elicited while palpating the mandibular rami. The myringotomy tubes were intact without and significant drainage. The nasal mucosa appeared normal without any evidence of erythema, epistaxis, or discharge. The rest of the physical examination was unremarkable. The laboratory data are shown in Table 1.

Urinalysis showed trace proteinuria and no RBC cast. Electrocardiogram and a chest X-ray were normal. She underwent a CT scan of her neck with contrast which was unremarkable except left maxillary sinus thickening. However, the apical part of the lungs showed multiple nodules bilaterally. A dedicated high-resolution CT scan of the chest revealed multiple bilateral nodules, 5–11 mm in diameter. The largest nodule was noted in the lingula that measured 11 × 9 mm. There was also evidence of pericarditis and small pericardial effusion. Given her long standing history of uncontrolled asthma, upper airway symptoms, eosinophilia, and multiple pulmonary nodules, a clinical diagnosis of EGPA was made and the patient underwent an extensive rheumatologic workup which is shown in Table 2.

**Figure 1:**
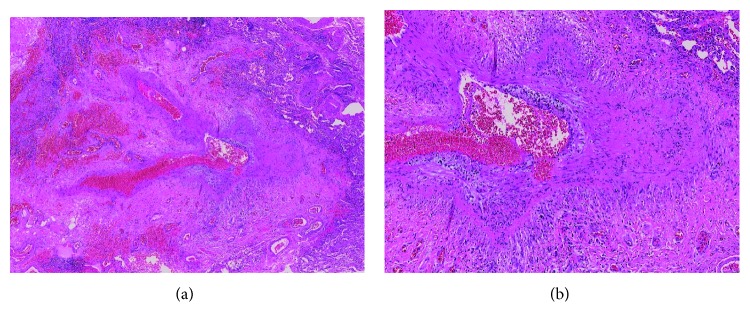
(a) Large pulmonary arterial branch with fibrinoid necrosis, granulomatous inflammation, and eosinophil-rich infiltrate (H&E stain, 40x). (b) Higher power demonstrating eosinophil-rich inflammatory infiltrate associated with fibrinoid necrosis (H&E stain, 100x).

**Figure 2:**
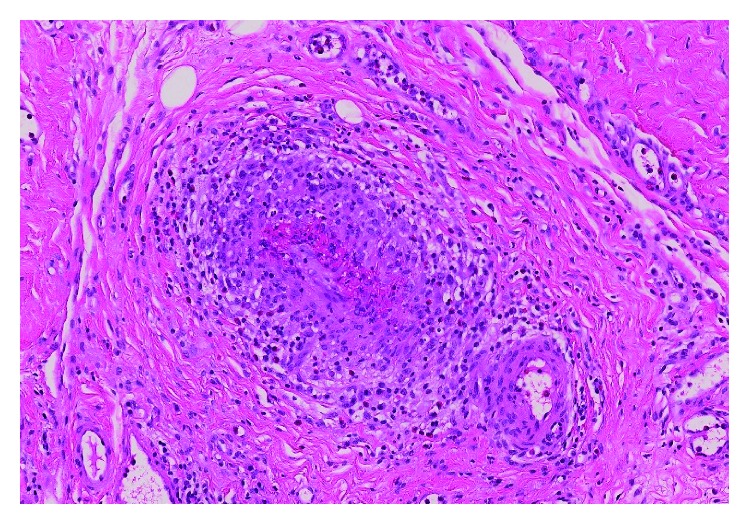
Small pericardial artery with vasculitis including numerous eosinophils (H&E stain, 200x).

The patient underwent an open lung biopsy of the lingular nodule as well as a pericardial biopsy. The histopathology was consistent with necrotizing granulomatous vasculitis with extravascular eosinophilic infiltrate. The histopathology slides are shown in Figures 1 and 2. The diagnosis of EGPA was confirmed.

The patient was started on high-dose prednisone and azathioprine in March 2009. She initially did well, but in August 2009 the patient was admitted to the hospital with pulmonary edema secondary to heart failure with reduced ejection fraction (HFrEF). The echocardiogram showed an EF of 25–30% with biventricular dilatation and global hypokinesis. No wall motion abnormality was identified. Cardiac catheterization was negative for coronary artery disease. No endomyocardial biopsy was performed. Given the high incidence of cardiac involvement in EGPA and a negative coronary angiogram, the myocardial dysfunction was thought to be secondary to EGPA and she was started on IV cyclophosphamide. Over the course of next 6 months, the patient completed 6 cycles of IV cyclophosphamide and started on mycophenolate. Steroid taper was continued. Her EF recovered completely, but she suffered from multiple vertebral body compression fractures and developed cushingoid appearance secondary to chronic steroid therapy. Between September 2009 and July 2011, she suffered from 2 episodes of sinusitis which was treated with short course of antibiotic. Her disease remained well controlled with normal inflammatory markers.

**Figure 3:**
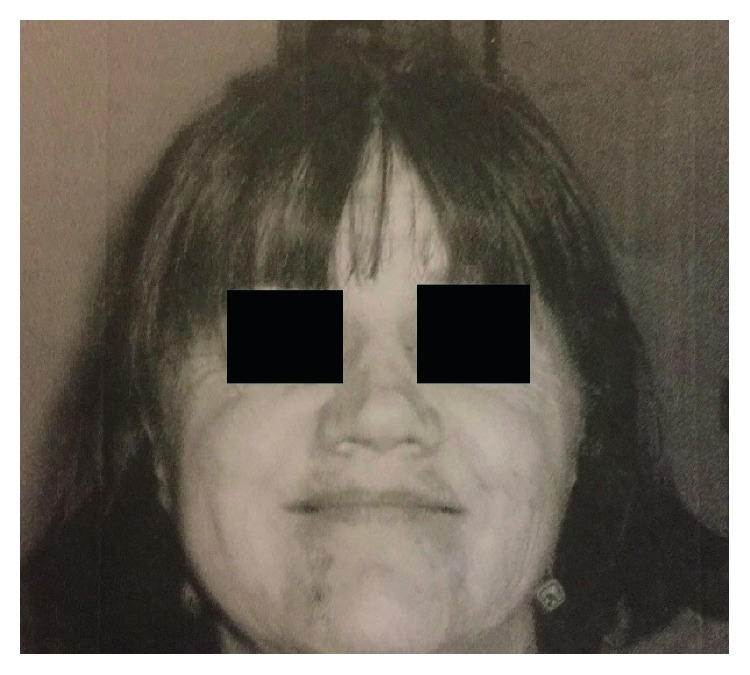
Saddle nose deformity.

During an office visit in July 2011, the patient was noted to have depressed nasal ridge (Figure 3). Physical examination showed nasal mucosal erythema. The patient underwent reconstruction of her nasal bridge in July 2013. Since 2011, the patient has been managed with mycophenolate without any incidence of exacerbation and is doing well currently.

## 3. Discussion

Our case represents the first case of saddle nose deformity secondary to EGPA that was confirmed by lung and pericardial biopsy.

EGPA is one of the AAV syndromes that predominantly affect medium- to small-sized vessels. Although EGPA can involve any organ system, upper airway, peripheral nerves, skin, and lungs are most commonly involved [2]. Ear, nose, and throat (ENT) involvement is manifested by allergic rhinitis, recurrent sinusitis, otitis media, nasal obstruction, and nasal polyposis [3]. Clinically, EGPA is characterized by long-standing history of rhinosinusitis, difficult-to-control asthma, and peripheral eosinophilia [4]. Histopathology in the vasculitic phase of the disease reveals eosinophilic infiltration of the vessel walls and necrotizing eosinophilic granuloma in extravascular tissue [5].

Destructive upper airway disease, such as saddle nose deformity (SND), is a well-known manifestation of AAV syndromes, especially with GPA [6]. Other causes of SND include relapsing polychondritis (RP), granulomatous infection, lymphomatoid granulomatosis, lymphoma, carcinoma, and trauma. Although significant ENT symptoms can be present in any of the AAV and contribute to patients suffering, destructive upper airway disease is a classic manifestation of GPA [6]. ENT involvement is seen in up to 90% of patients with GPA during the course of their disease [7]. Nasal septal perforation, mucosal ulceration, and collapse of the nasal bridge leading to SND are well-known complications of GPA [7]. The diagnosis of GPA is often delayed in patients with nonerosive ENT symptoms as they are initially mistakenly attributed to allergic etiology before an extensive workup is undertaken.

ENT involvement is common with EGPA, and paranasal sinus abnormalities have been included as one of the diagnostic criteria proposed by American College of Rheumatology [8, 9]. In a large observational French study that retrospectively reviewed 383 patients with EGPA diagnosed between 1957 and 2009, 48% of patients were found to have ENT involvement on presentation. The most common symptoms included rhinitis, nonerosive sinusitis, and nasal polyposis. Destructive upper airway manifestations, such as SND or septal perforation, were not present in any of the patients. 60% of patients with ENT involvement were found to be ANCA positive [2]. According to five-factor scoring (FFS) system, presence of ENT symptoms is considered to convey better prognosis [10].

The presence of SND in our patient is an intriguing clinical manifestation that has never been reported in patients with EGPA. Her other symptoms including difficult-to-control asthma over many years, chronic rhinosinusitis, otitis media, eosinophilia, and pulmonary infiltrate are consistent with typical progression of EGPA that was eventually confirmed by a lung biopsy. Why she suffered from erosive ENT disease is unknown.

Historically, GPA has been associated with presence of cytoplasmic-ANCA (C-ANCA), while MPA and EGPA have been characterized by perinuclear-ANCA (P-ANCA) positivity on immunofluorescence staining of ethanol-fixed human leukocytes [7]. With the advent of enzyme-linked immunosorbent assay (ELISA), most C and P-ANCA autoantibodies have been identified as anti-proteinase 3 (PR3) and anti-myeloperoxidase (MPO) antibodies, respectively. Thus, GPA is typically positive for PR3-ANCA, whereas MPA and EGPA are positive for MPO-ANCA. However, some patients with GPA and a positive C-ANCA have anti-MPO antibody and patients with EGPA or MPA are positive for P-ANCA with anti-PR3 antibody. The rate of ANCA positivity varies based on the type of AAV. In EGPA, the ANCA is positive in about 50% of patients [7]. The severity of AAV has been proposed to be related to the type of autoantibody rather than the individual vasculitic syndrome [11]. In general, presence of PR3-ANCA is considered to be associated with worse disease severity and more relapse [11].

Our patient suffered from severe EGPA with cardiac, lung, and upper airway involvement. She was treated initially with glucocorticoid and azathioprine. Subsequently, she required IV cyclophosphamide therapy for worsening disease. It is possible that the SND was a manifestation of severe systemic inflammation and developed slowly over a 2-year period, although the inflammatory markers were normal when SND was first observed. Even in patients with severe disease, SND has not been reported in the past. Presence of both MPO and PR3-ANCA together has been reported in patients with anti-GBM antibody disease [12] and users of cocaine contaminated with levamisole [13]. The patient was checked for PR3-ANCA positivity many times over the years, but there was no evidence for overlap syndrome. The other possibility would be lack of reporting due to attribution of SND to other causes.

## 4. Conclusion

Erosive upper airway disease from EGPA is unheard of and has never been reported. The knowledge regarding the presence of autoantibodies in AAV and their roles is disease causation, and manifestation is continuously evolving. We are reporting the first case of SND from EGPA with the hope to enrich the medical literature and encourage further research into identification of different disease phenotypes and move forward towards individualized management of these complicated life-threatening diseases.

## Figures and Tables

**Table 1:** Admission laboratory values.

Laboratory test	Result	Normal value
WBC	20.4	4.1–9.3 × 10^3^/*μ*L
Neutrophil (%)	70	41–67
Lymphocyte (%)	11	28–42
Eosinophil (%)	12	0–5
Absolute eosinophil	2492	50–500/*μ*L
Hemoglobin	9.7	11–14.7 gm/dL
Hematocrit	28.5	33–44
Platelet	589	130–350 × 10^3^/*μ*L
Electrolytes	Within normal range	—
BUN	5	7–21 mg/dL
Creatinine	0.7	0.7–1.2 mg/dL
Albumin	1.8	3.5–5.2 gm/dL
Total protein	6.3	6–8 gm/dL
Total bilirubin	0.9	0.1–1.2 mg/dL
ALT	67	5–60 IU/L
AST	81	5–45 IU/L
Alkaline phosphatase	179	30–115 IU/L
CRP	187	<8 mg/dL
ESR	115	0–25 mm/hour

**Table 2:** Rheumatologic workup.

Laboratory test	Result	Normal value
ANA	Negative	<320
Cytoplasmic ANCA	<20	<20
Perinuclear ANCA	40	<20
Atypical ANCA	<20	<20
Anti-myeloperoxidase antibody	49.1 H	<20
Anti-proteinase 3 antibody	2.3	0–3.5
Rheumatoid factor	142 H	<20
Anti-CCP	<15	<20
Anti-cardiolipin IgG, IgA, IgM	<15	<20
Beta-2 glycoprotein IgG, IgA, IgM	<9	<20
CH50	55	22–60
C3	147	70–163
C4	23.3	12–51
Anti-SSA	10	0–99
Anti-SSB	9	0–99
Anti-Jo	<0.2	0.0–0.9
Angiotensin converting enzyme (ACE)	20	12–68
Hepatitis panel	Negative	Negative
Anti-streptolysin O	44	<125
